# Stabilizing high-humidity perovskite solar cells with MoS_2_ hybrid HTL

**DOI:** 10.1038/s41598-023-39189-0

**Published:** 2023-07-25

**Authors:** Puteri Nor Aznie Fahsyar, Norasikin Ahmad Ludin, Noor Fadhilah Ramli, Puteri Intan Zulaikha, Suhaila Sepeai, Ahmad Shah Hizam Md Yasir

**Affiliations:** 1 Clean Technology Impact Laboratory, Taylor’s University, Selangor, Malaysia; 2 Solar Energy Research Institute, University Kebangsaan Malaysia, Selangor, Malaysia; 3 Faculty of Resilience, Rabdan Academy, Abu Dhabi, UAE

**Keywords:** Energy science and technology, Nanoscience and technology, Physics

## Abstract

The obstacle to the industrialization of perovskite solar cells (PSC) technology lies in their stability. This work rationalizes the PSC design with the employment of 2D-MoS_2_ as the hybrid hole transport layer (HTL). MoS_2_ was selected due to its unique optoelectronic and mechanical properties that could enhance hole extraction and thus boost the performance and stability of PSC devices. Five concentrations indicated MoS_2_ nanosheets were directly deposited onto the perovskite layer via the facile spin coating method. The electrochemical exfoliation and liquid exchange methods were demonstrated to obtain the lateral size of MoS_2_ nanosheets and further discussed their microscopic and spectroscopic characterizations. Remarkably, the optimum thickness and the excellent device increased the stability of the PSC, allowing it to maintain 45% of its degradation percentage ($\frac{\Delta PCE}{PCE}$) for 120 h with high relative humidity (RH = 40–50%) in its vicinity. We observed that lithium-ion can intercalate into the layered MoS_2_ structure and reduce the interfacial resistance of perovskite and the HTL. Most importantly, the 2D-MoS_2_ mechanism’s effect on enabling stable and efficient devices by reducing lithium-ion migration in the HTL is demonstrated in this work to validate the great potential of this hybrid structure in PSC applications.

## Introduction

Currently, the power conversion efficiency (PCE) of perovskite solar cells (PSC) has surpassed 25.7%, proving the enormous potential of this technology in the photovoltaic industry^1^. However, the stability issues of PSC devices that are mainly caused by humidity, thermal instability, UV light, and other factors greatly limit their commercialization^2–6^. In this context, for PSC devices to be commercially available in the market, they should at least have operational stability which is evaluated by the lifetime of PSC operation under 1-sun illumination or a certain bias condition that replicates the intrinsic stability of the device. Specifically, the stability of solar cells must satisfy the testing standard set by the International Electrotechnical Commission (IEC 61215)^7,8^. Thus, many strategies have been reported to improve the stability of PSCs, providing guidance to future research on fabricating PSC devices^9^. The instability of conventional PSCs is attributed to the use of organic hole-transporting materials, such as 2,2′,7,7′-tetrakis(*N,N*-dipmethoxyphenylamine)-9,9′-spirobifluorene (spiro-OMeTAD), which require a doping process with hygroscopic materials to expand their charge transport properties^10–13^. The performance and stability deteriorate due to the movement of lithium ions from the spiro-OMeTAD solution to the perovskite absorber layer^14–16^.

An attempt to substitute spiro-OMeTAD as the hole transport layer (HTL) material with PTAA, NiO, PEDOT:PSS, and CuSCN has been reported, but the desired performance has not been achieved. PTAA is the closest competitor to replacing spiro-OMeTAD in most third-generation solar cells. However, structures using spiro-OMeTAD still dominated the highest PCE chart at 25.2% compared to only 21.2% for structures with PTAA^17^. Another approach is by integrating a buffer layer to improve the interfacial perovskite/HTL losses. By reducing shunting degradation, a layer called a buffer acts as an effective barrier between the metal electrode (gold or silver) and the perovskite layer^18–21^. The buffer layer is divided into two categories namely the passive layer and the active layer. In this context, the passive buffer layer indicates that the layer has only one role i.e. as a barrier layer of ions, thus intrinsic ions are incapable to penetrate other crucial layers. Meanwhile, the term active buffer layer refers to the capability of the layer to enhance the conductivity of the material used and the charge carrier in addition to acting as an ion transfer barrier.

Recently, two-dimensional transition metal dichalcogenide (2D-TMD) materials have gained importance due to their inimitable physical and chemical properties, such as solution processability, mechanical robustness, and tailored electrical conductivity, which are suitable for photovoltaic applications^22^. 2D-TMD materials, such as MoS_2_, MoSe_2_, WS_2,_ and TiS_2_, are currently used as an interlayer in the PSC structure, normally sandwiched between the perovskite and the electron or hole-transporting layer. The materials show that the energy band can be aligned to the perovskite active and transporting layers, prevent shunt contact formation, improve the grain size and the crystalline quality, avoid direct hydrophilic contact interface, and thus lead to increased efficiency and stability^23–28^. Specifically, many attempts have been made to use MoS_2_ for the same purpose since 2016. For instance, Capasso et al. employed MoS_2_ flakes in the n-i-p structure with augmented stability of up to 550 h, but the PCE decreased slightly^24^. Then, Jiang et al. successfully developed a flower-like MoS_2_ microstructure that acts as a powerful sorbent to lithium ions in spiro-OMeTAD^26^. In the latest work of Liang et al., different procedures were analyzed to incorporate MoS_2_ into the perovskite to attain an optimized structure to increase the PCE and the stability after 1 h^28^. The incorporation of MoS_2_ into PSCs provides a new way of attaining increased solar cell efficiency and long-term stability. However, additional investigations on MoS_2_ are required to explore the maximum potential of this material^29,30^.

In this work, we propose the usage of MoS_2_ flakes as a hole transport material and the combination of the hole extraction interlayer with spiro-OMeTAD. The MoS_2_ flakes were prepared using liquid phase exfoliation and dispersed in an IPA solvent for use as an active buffer layer. Several MoS_2_ concentrations were prepared homogenously and deposited between the perovskite and spiro-OMeTAD to investigate their ability to control the photooxidation, moisture, and chemical processes. The samples are denoted as M0, M1, M2, M3, and M4, indicating the MoS_2_ layer and the concentration (e.g., M1 is for MoS_2_ with a concentration of 1 mg mL^−1^, while M0 is for the concentration of 0 mg mL^−1^ or the reference cell). Although numerous structures with MoS_2_ have been reported in the literature on PSC, we highlighted the importance of finding the optimal MoS_2_ concentration to conceal the defects on the perovskite surface layer. In this work, the mechanism was observed to associate the thickness of the MoS_2_ with its concentration. To obtain more insight into the influence of MoS_2_ layer addition, the material properties and performance were investigated. This is the first report to clarify the significance of the MoS_2_ concentration in hybrid-HTL-based PSC devices. This study provides fresh explanations with a focus on structure, morphologic tuning, and performance to enhance the PCE and stability of PSC devices. This contribution provides new insights into physical, morphological, optical, and material-level performance to improve the device’s PCE and stability.

## Experimental setup

### Solar cell fabrication

Firstly, the FTO (15 Ωsq^−1^ from Solaronix) substrates were etched with zinc powder and HCl then cleaned in an ultrasonic bath for 10 min each using acetone, ethanol, and IPA. A compact TiO_2_ layer (bl-TiO_2_) was deposited on the substrate by spin-coating (3000 rpm, 30 s) of 1 mL titanium isopropoxide in 1 mL ethanol. A mesoporous TiO_2_ (mp-TiO_2_) was diluted with absolute ethanol with a 1:9 ratio and coated onto FTO/bl-TiO_2_ substrate by spin-coating (4000 rpm, 20 s)^31^. The substrates were sintered on the hotplate at 450 °C and 45 min. 553 mg PbI_2_ (99%) was mixed in 1 mL DMF (99.8%) and 100 μL tBp, then 30 mg CH_3_NH_3_I in 1 mL IPA. 70 μL of PbI_2_ precursor solution was spin-coated on the mp-TiO_2_/bl-TiO_2_/FTO substrate at 3000 rpm, 60 s, and heated at 70 °C for 30 min. 200 μL of MAI precursor solution was deposited on the substrate then spin-coated at 3000 rpm, 20 s and heated at 95 °C for 30 min. Subsequently, 100 μL of MoS_2_ dispersion with different concentrations was spin-coated on the perovskite thin films at 2000 rpm, 45 s, and annealed at 70 °C for 1 min. The HTL was prepared by mixing 1 mL spiro-OMeTAD solution (72.3 mg in 1 mL chlorobenzene), 17.5 µL Li-TFSI solution (520 mg Li-TFSI in 1 mL acetonitrile), and 28.8 µL tBP. After cooling, 50 µL of the spiro-OMeTAD precursor was dropped on perovskite/mp-TiO_2_/bl-TiO_2_/FTO substrate and then spin-coated at 4000 rpm for 20 s. All chemicals were purchased from Sigma Aldrich. Finally, the device was completed with the silver (Ag) top electrode which was deposited via thermal evaporation. The fabrication process was conducted in controlled relative humidity (RH = 40–50%) in the glove box. The devices are unencapsulated with a cell active area of 0.07 cm^2^.

### Characterization

The MoS_2_ dispersions were characterized by optical absorption spectroscopy in the range 300–900 nm with a Lambda 35 Perkin Elmer UV–Vis spectrophotometer. Raman spectroscopy (Thermo Scientific DXR2xi) was performed on the MoS_2_ flakes collected from the dispersions. The transmission electron microscope (FEI Talos, L120C) was used in analyzing the crystal structure and composition of MoS_2_ dispersion. The perovskite surface morphology and cross-sectional images were characterized by FESEM (ZEISS, Merlin Compact), and Nanosurf Easyscan2 AFM. XRD spectra were carried out by using the X-ray diffractometer model Bruker D8 advance operated at a 2θ angle. Solar simulated AM 1.5G sunlight was employed with a solar simulator calibrated to give 100 mW/cm^2^ using a standard Si photovoltaic cell (Daystar Meter) for the PSC. *J–V* curves were recorded with a Keithley 2400 source meter under the scan rate was set to 0.1 V s^−1^. The electrochemical impedance spectroscopy (EIS) measurements of PSC devices were analyzed at 100 mW cm^−2^ illuminations with 0.5 V bias. All procedure was carried out in ambient air without any humidity control (RH = 40–50%) and kept unencapsulated.

## Results and discussion

**Figure 1:**
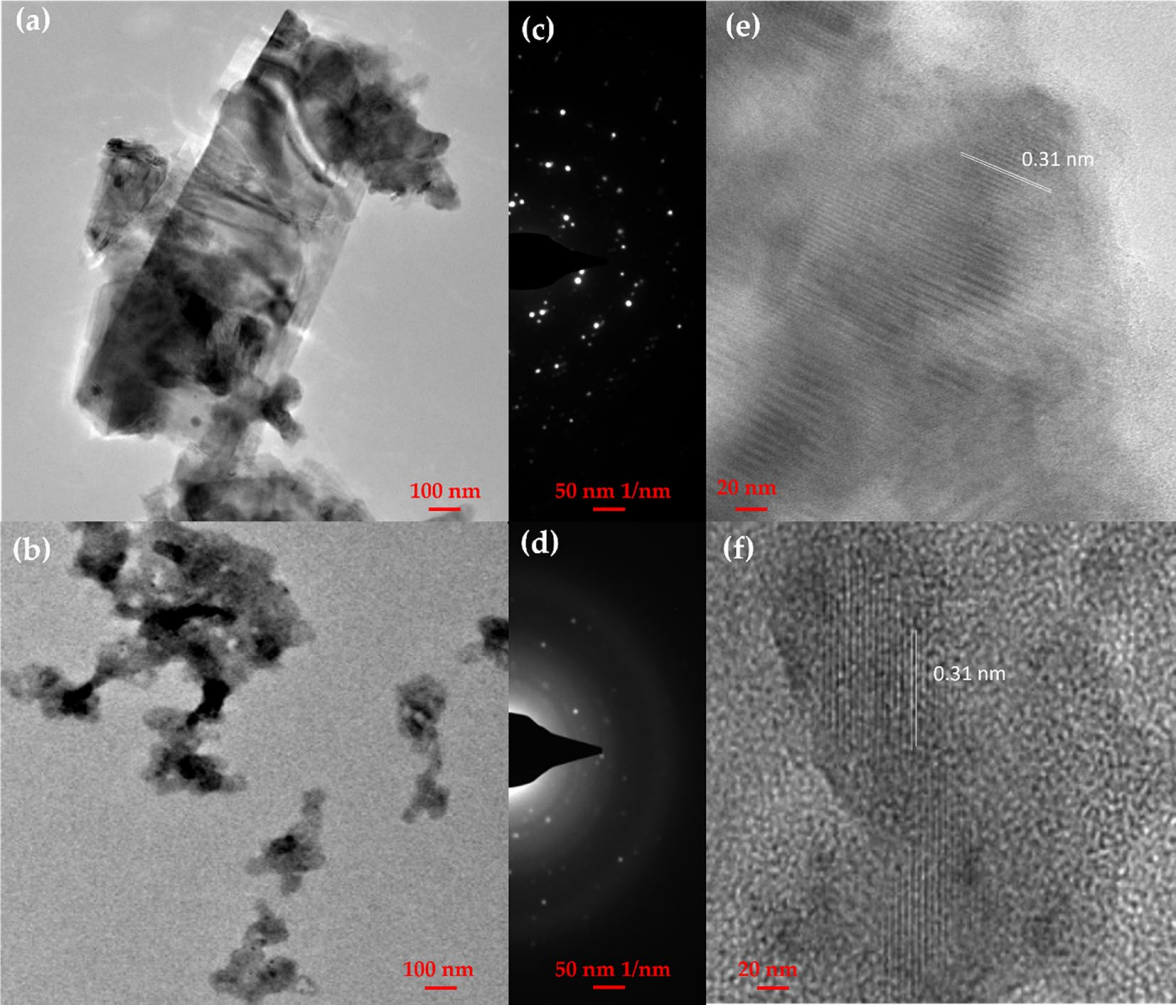
(**a**,**b**) TEM images of exfoliated MoS_2_ in NMP and IPA, respectively; (**c**,**d**) the corresponding electron diffraction pattern for NMP and IPA, respectively; and (**e**,**f**) exhibit the periodicity of the Mo and S atomic columns in NMP and IPA, respectively.

First, an exfoliated MoS_2_ is attained by sonication via liquid exfoliation and solvent exchange process in NMP as shown in Fig. S1. To ensure less impairment onto the perovskite thin film surface, this facile method has been widely used in the n-i-p structure of PSC due to the low annealing temperature required^20,24,32^. The surface energy of NMP (~ 0.041 N m^−1^ compared to IPA which is around ~ 0.022 m^−1^ at 25 °C) is suitable to break a weak van der Waals bond of layered MoS_2_. To diminish solvent degradation and heating, ultra-sonication temperature has not exceeded 70 °C with 10 min rest until 8 h. The MoS_2_ dispersion is centrifuged at 4000 rpm for 20 min to eliminate unexfoliated residue. Then, 90% of the supernatant was collected and undergo a solvent exchange process to optimize the NMP elimination. After the processes, we obtained the MoS_2_ yield weight of 32.2 mg after processes from the initial weight, of 100 mg**.** Then, the MoS_2_ yield was re-dispersed in 10.0 mL of IPA with a concentration of 10.0 mg mL^−1^. An absorbance spectrum of exfoliated MoS_2_ in NMP and IPA is shown in Fig. S2. Two peaks detected at 680 nm and 620 nm were featured for IPA due to excitonic transition^20,32^. Then, the first and second onset peaks were observed at 700 nm and 460 nm, respectively^33^. The MoS_2_ has diluted in NMP and IPA with 1.0 mg mL^−1^ concentration. The TEM images of a MoS_2_ dispersion in NMP and IPA are shown in Fig. 1a,b, respectively. The corresponding electron diffraction pattern is shown in Fig. 1c,d which indicate the sixfold symmetry. The atomic column of Mo and S are visible, and the distance of lattice planes is 0.31 nm for both NMP and IPA as shown in Fig. 1e,f. The results demonstrate there is no physical and chemical structure alteration after the solvent exchange process.

**Figure 2:**
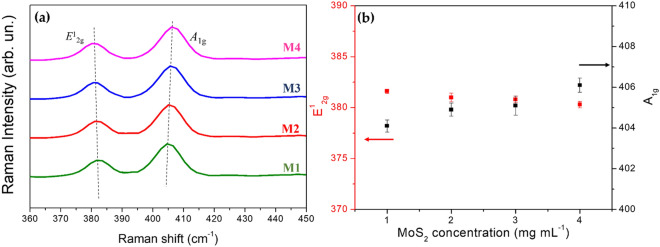
(**a**) Raman spectra of M1–M4 samples cast on Si/SiO_2_, (**b**) E^1^_2g_ and A_1g_ modes extracted from (**a**).

Raman spectroscopy is a powerful non-destructive characterization tool that can detect first-order Raman active modes. In the layered structure of MoS_2_, there are two vibration modes i.e. vibration within the layer (intralayer) and pattern produced by the movement of the complete layer (interlayer). These two modes can be used to determine the number of MoS_2_ as many reports previously^34,35^. The in-plane E^1^_2_g mode resulted from the contrary vibration of two S atoms concerning the Mo atom, while the A_1g_ mode is linked with the out-of-plane vibration of only the S atom in the opposite direction. The Raman spectra of MoS_2_ excited by 532 nm laser line in Fig. 2a shows individual mapping corresponding to the peaks of different layer numbers, which are represented by different MoS_2_ concentrations which vary from 0 to 4.0 mg mL^−1^. As the increase of the concentration, the pattern of E^1^_2_g is ~ 381.6 cm^−1^ shifts move towards lower frequencies, and the pattern at A_1g_ is 404.6 cm^-1^ shifts move towards higher frequencies. The detail values for Raman peak shifts tabulated in Table S1 and Fig. 2b exhibit the frequencies of E^1^_2g_ and A_1g_ modes extracted from Fig. 2a.

Comparably, similar peaks for bulk MoS_2_ within the range of 375–390 cm^−1^ for E^1^_2_g and 403–408 cm^−1^ for A_1g_ were found in the previous report via experimental and density functional theory (DFT)^24,34–36^. The frequency alteration between E^1^_2g_ and A_1g_ is considered the most effective indicator of the number of layers. This work’s number of layers increases proportionally to the MoS_2_ concentration. Indeed, from M1 to M4 samples as depicted in Fig. 2a, the calculated frequency difference increases monotonically from 23 to 27 cm^−1^, indicating reliability and confirmation as a thickness indicator^37^. For the mechanism sage, the shift of A_1g_ parallel with the thickness increment is due to interlayer interaction enhancement. The effective restoring forces are enhanced on the atoms, whereas more unanticipated drops of E^1^_2g_ with thickness increment have been ascribed to the cumulative dielectric screening of the long-range Coulomb forces which declines the overall reinstating energy on the atoms.

**Figure 3:**
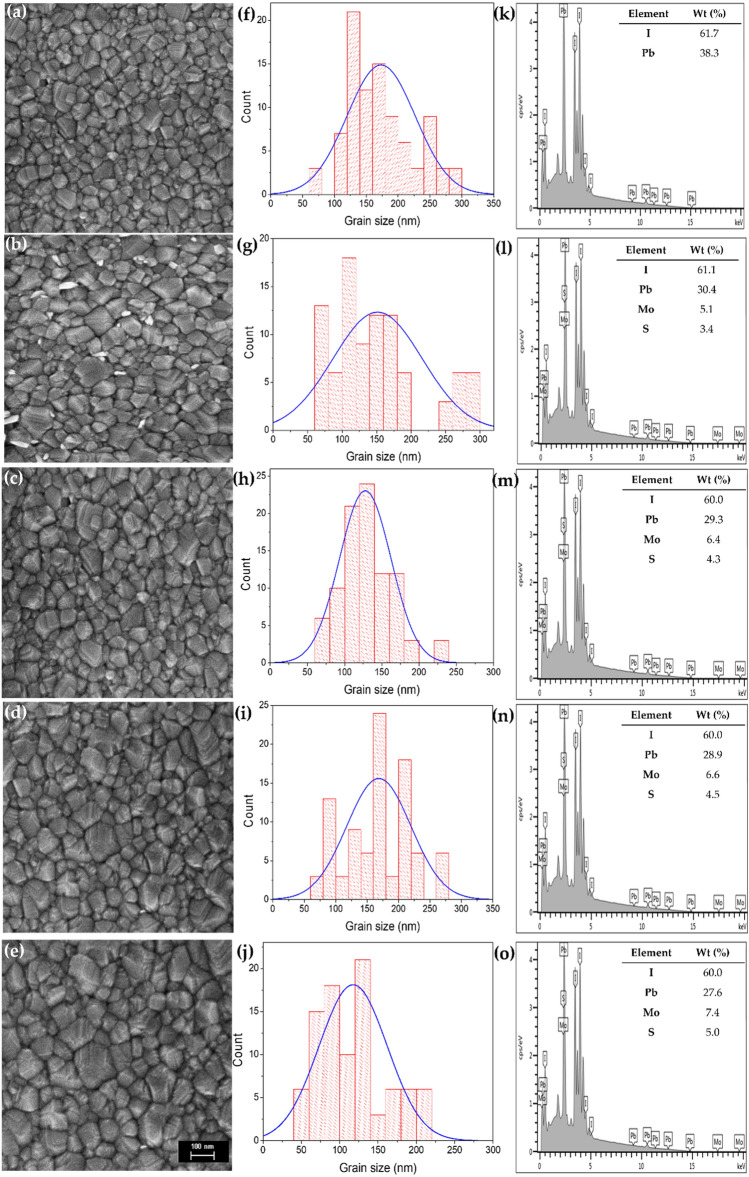
(**a**–**e**) FESEM morphology images for M0–M4 samples, (**f**–**j**) the particle size distribution for M0–M4 samples, respectively, and (**k**–**o**) FESEM-EDS for M0–M4 samples, respectively (and the corresponding element and weight percentage).

The topographical information obtained from FESEM surface images then confirmed the morphology of respective samples in Fig. 3a–e. With the naked eye, it can be seen the surface of perovskite/MoS_2_ thin films turned greyish in the presence of MoS_2_. The pinholes were hypothetical to accelerate the internal diffusion of the gas molecules in the surrounding air. This phenomenon possibly will have unanticipated consequences for the perovskite layer. Furthermore, because this condition arose in a rapid period, the perovskite film would face deterioration and become degraded because of the chemical compound’s superficial diffusion. As the concentration varied, the thickness of MoS_2_ was recorded to be 9 nm, 29 nm, 45 nm, and 65 nm for M1, M2, M3, and M4, respectively. The sub-layer thicknesses of PSC are the TiO_2_ layer (155 nm), CH_3_NH_3_PbI_3_ (363 nm), and spiro-OMeTAD hybrid MoS_2_ (270 nm)^38–40^. MoS_2_ has multiple functions and can be used as a hole accumulator of spiro-OMeTAD to boost hole mobility and improve film conductivity. In addition, MoS_2_ acts as a strong adsorbent for Li+ ions, which expands the device stability of the amorphous spiro-OMeTAD organic thin film^26^. Figure 3f–j correlated to the particle size distribution. The presence of MoS_2_ does not indicate any deviations in the grain size, as proved by the average grain size for the samples distributed similarly from 50 to 200 nm. Next, the FESEM-EDS analysis confirmed the presence of elemental composition in the thin film as shown in Fig. 3k–o. A slight difference in the weight percentage for perovskite and MoS_2_ elements can be observed. The increment in the percentage of Mo and S atoms is directly proportional to the increment of concentration.

**Figure 4:**
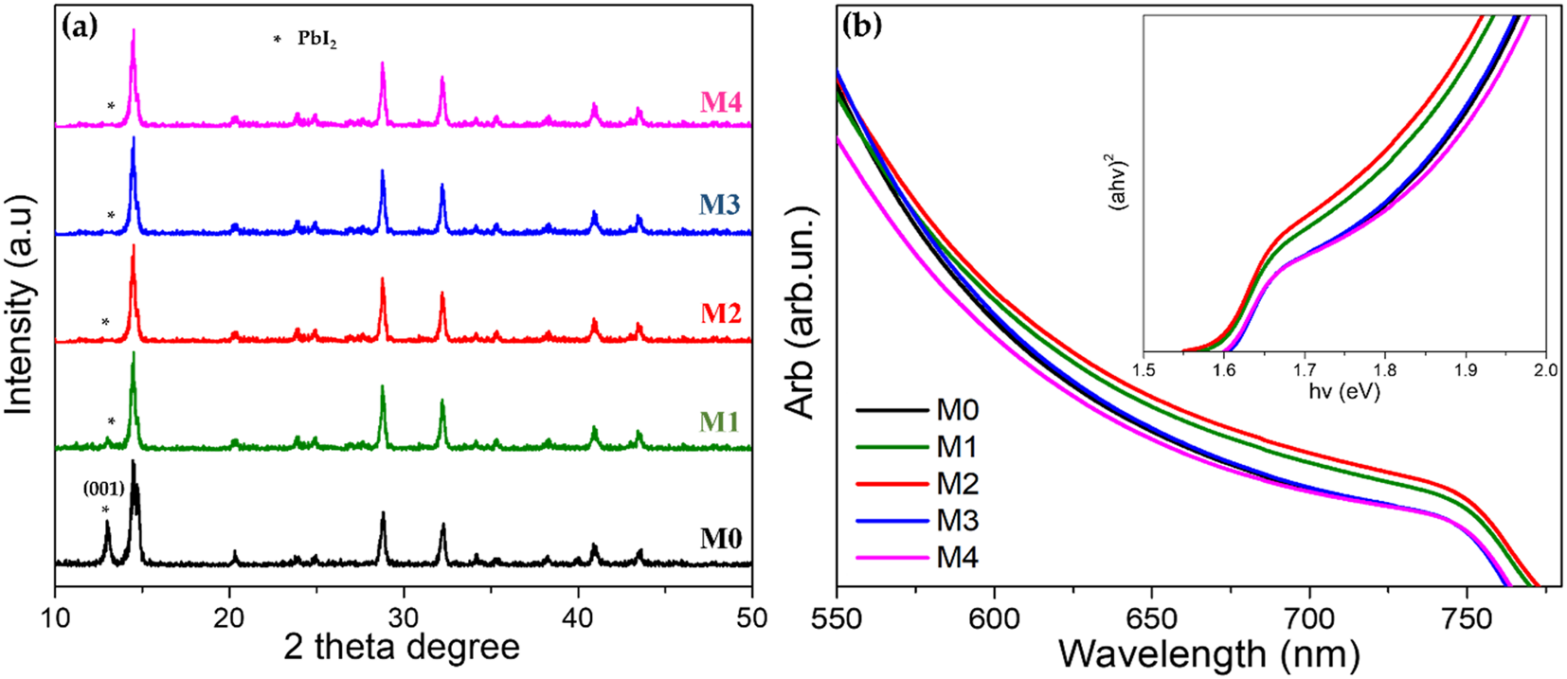
(**a**) XRD patterns for M0–M4 samples, and (**b**) absorbance spectra for M0–M4 samples prepared under (RH = 40–50%). The Tauc plot of each sample is plotted in the inset of (**b**).

To study the properties of the crystal structure of the MoS_2_ on top of the perovskite layer prepared at different concentrations, XRD patterns were taken immediately after the preparation of the films. As shown in Fig. 4a, multiple perovskite crystal characteristic peaks were observed. The sample for M0 indicates a small diffraction peak of PbI_2_ at 2θ = 12.8° which corresponds to the 001 facet, which is presumably due to perovskite degradation during the fabrication process^41^. The small peak declines for M1–M4 samples proportionally, which proves that the layer can repel moisture from being captivated into the other layers although the fabrication process is done under high humidity conditions (RH = 40–50%). The other characteristic peaks correspond well to MAPbI_3_ (at 2θ = 14.5° and 28.7°) for all samples with crystal size obtained ~ 31.9 nm^42,43^. In addition, some miscellaneous peaks were detected, which explained the presence of residual CH_3_NH_3_I will be decomposed and the product of I_2_. It is noted that no additional peaks related to MoS_2_ layers were observed due to the small amount of MoS_2_ layers present on top of the perovskite layer even for 4.0 mg mL^−1^ concentration which is in good agreement with FESEM investigation related to grain size.

UV–Vis analysis was carried out to investigate the significance of various MoS_2_ concentrations in PSC. According to the Tauc plot inset of Fig. 4b, the bandgap of 1.59 eV and 1.58 eV measured for the M0 sample (without MoS_2_) and M2 sample, respectively which is close to the 1.55 eV bandgap presented by a previous study^44^. For the M1, M3, and M4 samples, the bandgap of 1.59 eV, 1.60 eV, and 1.61 eV were recorded respectively. The band gap has been tabulated in Table S2. These concentrations of MoS_2_ have a slightly significant influence on the perovskite bandgap, however more or less it affects the light absorption of the device, as shown in Fig. 4b. The light absorption of M2 is the highest then reduced by the higher concentration, which agreed with the declines in PSC performance as well.

**Figure 5:**
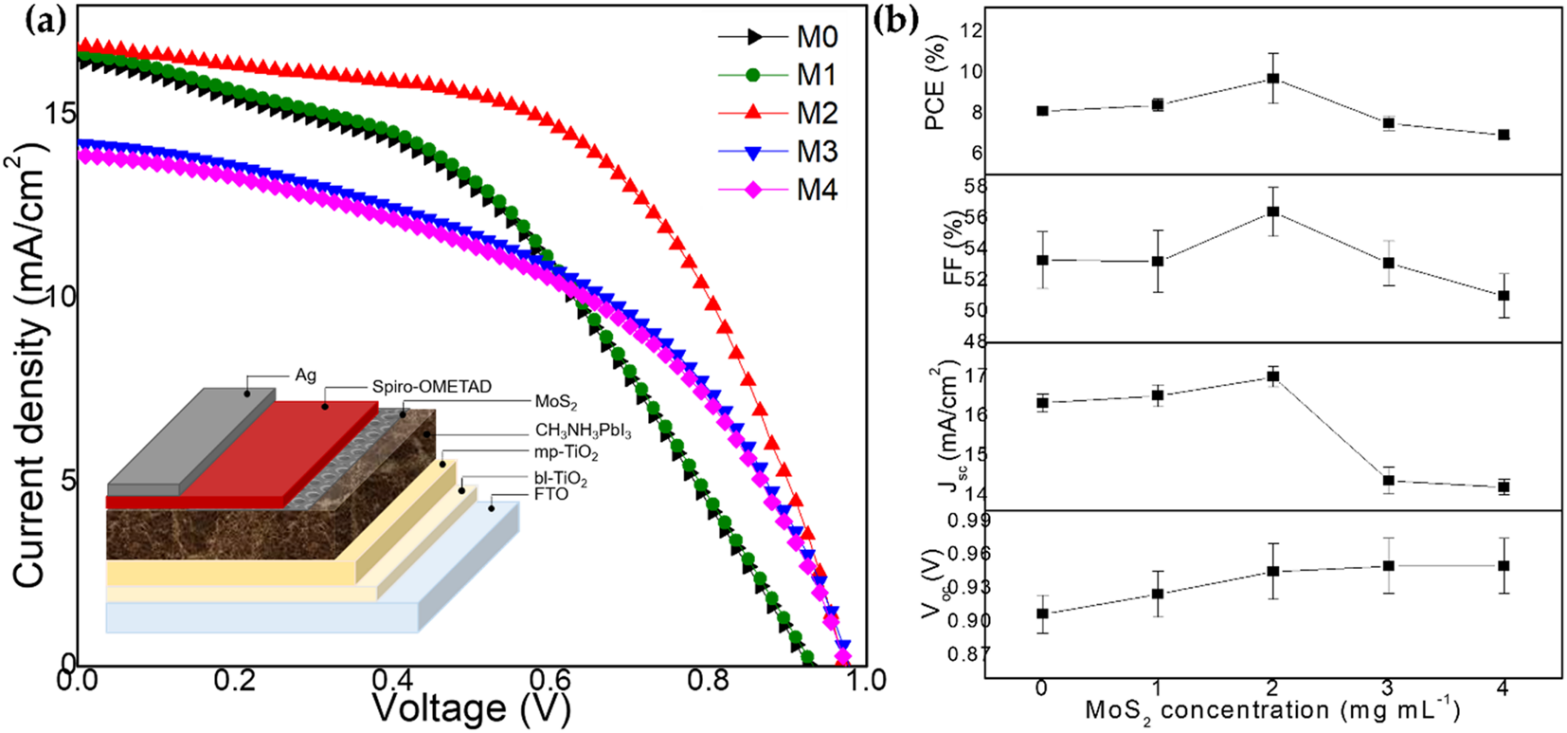
(**a**) *J*–*V* curve for M0–M4 samples. The device structure diagram is in the inset, along with (**a,b**) average photovoltaic parameters of at least 10 devices for M0–M4 samples.

**Figure 6:**
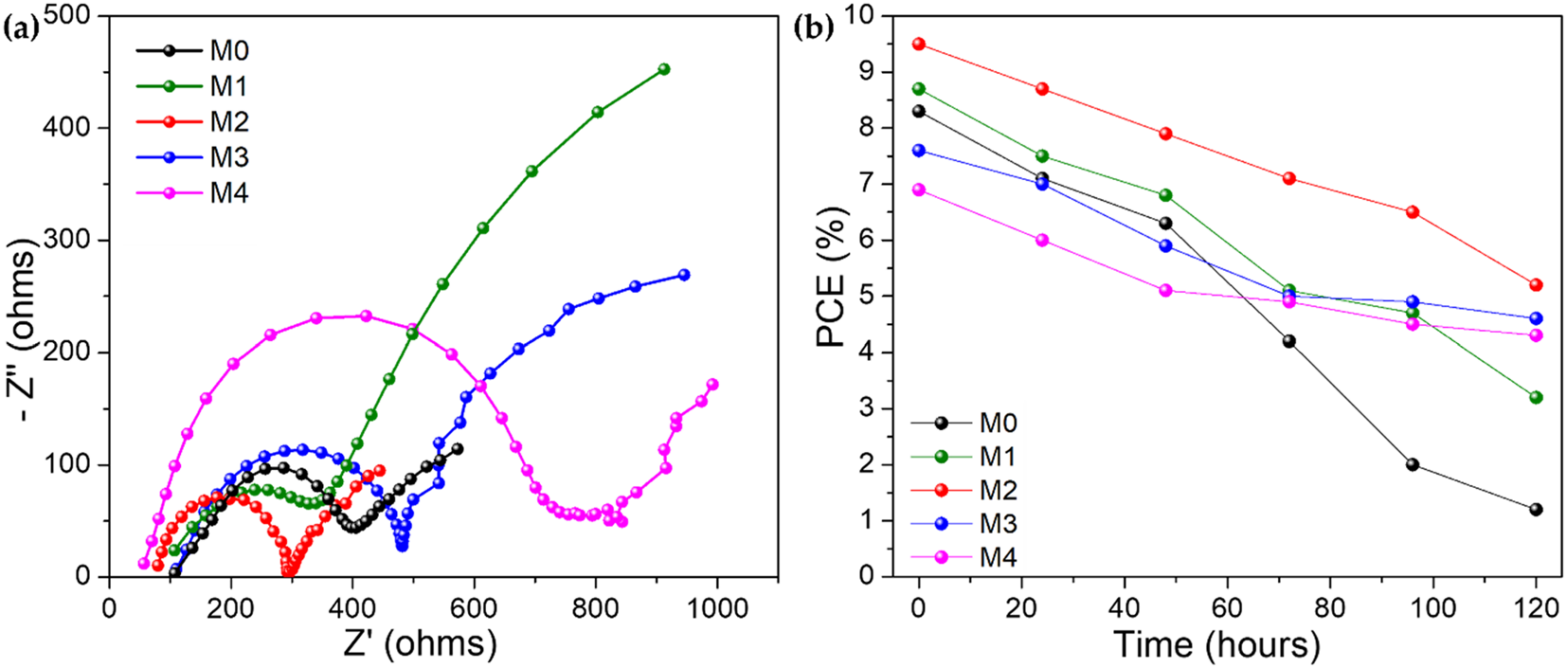
(**a**) EIS spectra for M0–M4 samples and (**b**) stability test for 120 h for M0–M4 samples.

To investigate the consistency, at least ten devices were prepared using similar process conditions for this study. Figure 5a depicts the photocurrent density–voltage (*J–V*) curves of PSC employing different MoS_2_ concentrations. Figure 5b depicts the statistic of the photovoltaic parameters of the corresponding 10 devices, as well as the trend of device performance, which corresponds to the data in Table 1. The charge transport and interfacial charge-transfer processes in TiO_2_/CH_3_NH_3_PbI_3_ and CH_3_NH_3_PbI_3_/MoS_2_/spiro-OMeTAD interfaces were evaluated using EIS measurements. Figure 6a shows the Nyquist plots of PSC at 0.5 V bias and the equivalent circuit of PSC. In the Nyquist plot, a series resistance (*Rs*) was ascribed to the FTO and a wire electrode with some other additional implements. The first semicircle arc in the high-frequency region represented the electron transport resistance (*Rct*) at the CH_3_NH_3_PbI_3_/MoS_2_/spiro-OMeTAD/Ag interface, meanwhile, the second semicircle arc in the low-frequency region represented the recombination resistance (*Rrec*) at the TiO_2_/CH_3_NH_3_PbI_3_ and FTO/TiO_2_, and one transmission line in the low-frequency corresponded to the Warburg impedance (*Zw*). The detail value for *Rct* and *Rrec is* summarised in Table S3. According to the Nyquist plot, the changes in both semicircle arcs were caused by changes in resistances in bulk PSC as well as the significant effect of MoS_2_ concentration variation. Furthermore, the character that exists in the low-frequency range could be assigned to the dielectric reduction process which is governed by an interfacial ion restructuring for instance happened in different cations in the perovskite structure. For this study, the hole-selective contact (MoS_2_/spiro-OMeTAD) and the electron-selective contact (TiO_2_) are the same for all samples, the observed systematic variation of *Rct* and *Rrec* values the retarded charge combination decreased dielectric reduction in the order M2 > M1 > M0 > M3 > M4, which consistent with the trend of PSC performance summarized in Table 1. The blending of MoS_2_ into perovskite had an effect both decreasing the rate of charge recombination and increasing *V*_*oc*_ value and slowing the dielectric relaxation which increased *J*_*sc*_ and *FF* value. Once the concentration was amplified to be 3.0 mg mL^−1^ and above, it led to inhomogeneity of film and poor *FF* thus reducing the overall performance of PSC.

**Table 1:** Photovoltaic-performance parameters for M0–M4 samples under 1 sun illumination (AM 1.5 G, 100 mW cm^−2^) for ten devices at reverse scan direction.

Sample	*J*_*SC*_ (mA/cm^2^)	*V*_*OC*_ (V)	*FF*	PCE (%)
M0	16.6	0.92	0.54	8.3
M1	16.8	0.94	0.54	8.5
M2	17.2	0.97	0.57	9.5
M3	14.5	0.97	0.53	7.6
M4	14.2	0.97	0.50	6.9

To associate the degradation process, the devices remained unencapsulated under an ambient environment (RH = 40–50%) for 120 h, and the measurement was taken every 24 h. Figure 6b presented the stability test for 120 h and the trend shows the degradation rate for sample M0 (without MoS_2_) dropped drastically while sample M2 shows the promising stability of the PCE. Meanwhile, the trend indicated the $\frac{\Delta PCE}{PCE}$ corresponding to MoS_2_ concentration, which the $\frac{\Delta PCE}{PCE}$ values are 85%, 63%, 45%, 39% and 37% for M0, M1, M2, M3, and M4 samples respectively. The observation was made, and the yellowish color of the sample started to appear. This is due to a few factors, such as exposure to moisture or other compounds. Besides, the Ag electrode corroded due to the Li+ ion residue still migrating to the perovskite layer. Table S4 shows significant deterioration in the *FF* for the sample without MoS_2_ due to the high revealing of moisture. Moisture will cause the electrode to rapidly degrade and corrode the silver layer on top of the spiro-OMeTAD film. Low *FF* values usually imply high series resistance, low shunt resistance, a high ideality factor, and a high reverse saturation current. Additionally, low shunt resistance is physically due to the partial bypass of the solar cell, while high series resistance is due to the HTL resistance and metal–semiconductor contacts. Whereas the high reverse saturation current is due to the high recombination in the active region. Detailed photovoltaic performance can be referred to in Tables S5–S8.

**Figure 7:**
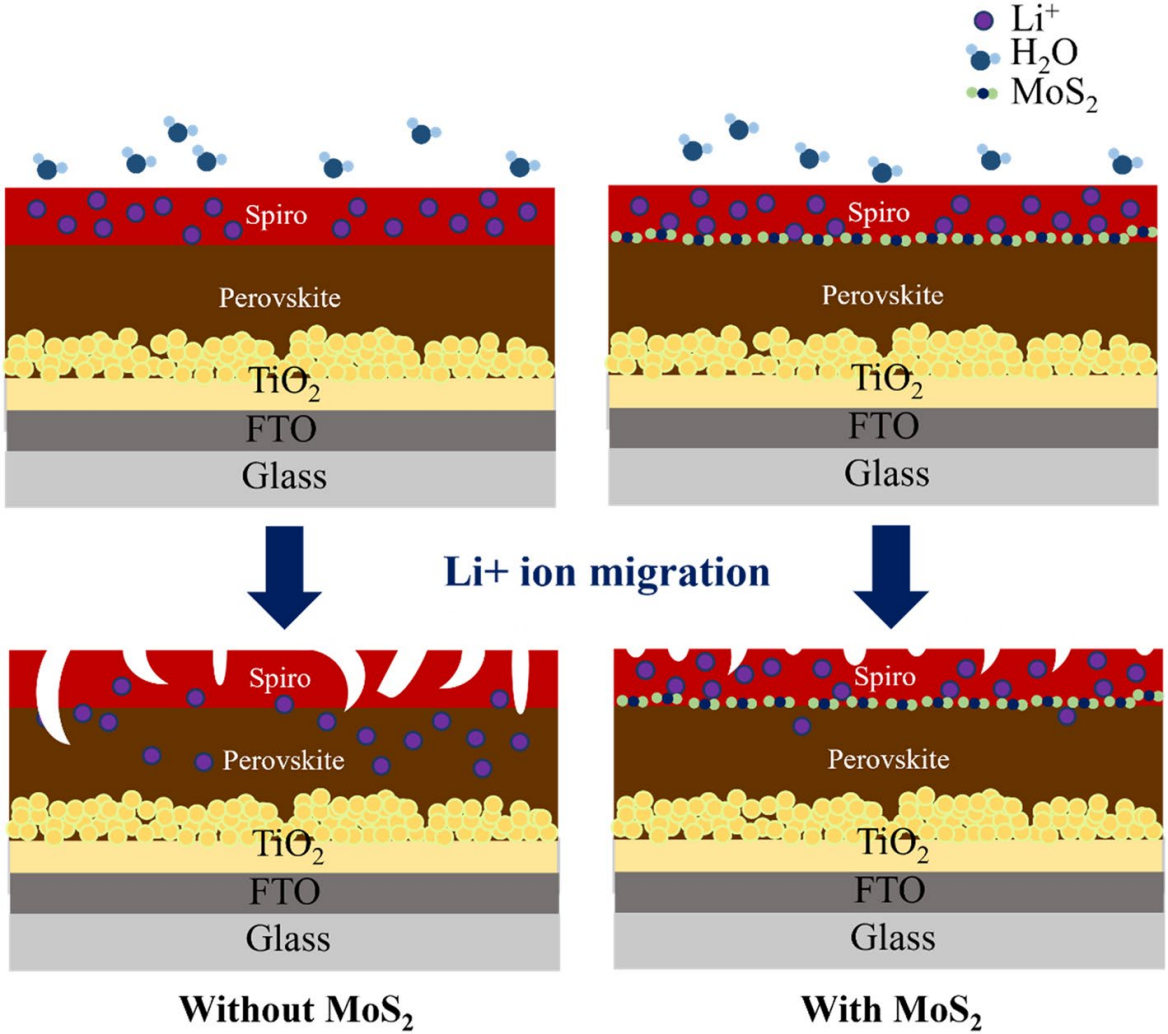
Schematic diagram of the mechanism by which MoS_2_ stifles the Li+ ions.

Finally, to gain an understanding of the mechanism, Fig. 7 depicts a schematic of the MoS_2_ stifling the migration of Li+ ions from the Spiro-OMeTAD layer to the perovskite layer. Moisture simply penetrates the perovskite layer through the invasion path of amorphous spiro-OMeTAD in the absence of MoS_2_. Similarly, the incorporation of the MoS_2_ layer, which managed to captivate and restrain the Li+ ion in the spiro-OMeTAD layer, prevents moisture from entering. This demonstrated that the presence of MoS_2_ could shorten the invasion path and limit Li+ ion migration from spiro-OMeTAD to the perovskite layer. As a result, the reason for the PSC’s improved stability can be attributed.

As validation, several previous studies using MoS_2_ as HTL have been tabulated as a comparison with this study. Firstly, Capasso et al. reported the stability of Spiro-OMeTAD-based PSC with 2D-MoS_2_ prepared via the LPE method and obtained 3 nm thickness. MoS_2_ nanosheets were deposited on top of the perovskite surface by the spin coating method in a mesoscopic PSC device. The reported PCE value as high as 13.3% was found to decrease up to 34% from the initial PCE value due to the inappropriate MoS_2_ thickness and forming an additional layer in the overall device^24^. Next, as HTL, PEDOT: PSS is frequently criticized for its intrinsic acidity and hygroscopic effects, which will affect the stability of PSC. Alternatively, water-soluble 2D-MoS_2_, 90 °C (a thin film of 11 nm thickness) was used as HTL in the p-i-n PSC structure. PCE values for HTL MoS_2_-based cells compared to PEDOT: PSS PSC based reported with 15.4% and 20%, respectively. This is due to the improved energy band orientation between MoS_2_ valance band and perovskite leading to higher *Voc*. Furthermore, the MoS_2_-based PSC device managed to maintain 78% of the original PCE value after 56 days in the glove box whereas the reference PSC device decreased to zero after 35 days^20^. As discussed, the use of spiro-OMeTAD should be supplemented with Li-TFSI and TBP dopants to enhance the hole mobility and conductivity of Spiro-OMeTAD films. However, the integration of this dopant in Spiro-OMeTAD will cause other problems such as hygroscopic properties and equilibrium, which are considered to be key factors for accelerating the deterioration of the Spiro-OMeTAD layer. In addition, the migration of Li+ ions from Li-TFSI solution is also an irritating problem and there is still no effective method to overcome it to date. Jiang et al. al. verified an effective method to improve the performance of PSC devices by altering the Spiro-OMeTAD coating with flower-like MoS_2_ nanoparticles^26^. The integration of MoS_2_ creates effective charge allocation with Spiro-OMeTAD molecules, enhanced film conductivity, and increased hole mobility in Spiro-OMeTAD films. The resulting PSC with a PCE rate as high as 20.18%. In addition, the incorporation of MoS_2_ nanoparticles enhanced the film stability of the Spiro-OMeTAD, resultant in highly prolonged cell stability by maintaining 86% original efficiency even after 300 h without encapsulation in air.

Recently, Liang et al. has introduced 2D-MoS_2_ nanoparticles of the buffer layer between the perovskite and HTL layers to improve the stability of metallic-halide organo PSC. The nanoparticles were obtained through an LPE approach, and a mesoporous-structured solar cell device and the FA_85_MA1_5_PbI_85_Br_15_ absorbent composition were fabricated^28^. PSC devices achieve a relative PCE of 14.9%, with a much longer lifespan stability than standard PSC. After 1 h, the PCE of the PSC device with the buffer layer managed to maintain 93.1% of the initial value, while the standard PSC dropped to 78.2% of the initial efficiency. Table 2 summarizes the PSC structure with MoS_2_ as HTL including this study. Comparably, this study highlights the incorporation of MoS_2_ as an HTL hybrid layer with optimal concentration values so that improved performance efficiency and device lifespan stability can be obtained. More importantly, the optimum MoS_2_ concentration and thickness could act as strong absorbers for Li+ ions, which can decelerate the migration of Li+ from Spiro-OMeTAD to the perovskite layer.

**Table 2:** Summary of MoS_2_ as HTL in PSC structure.

Structure	PCE (%)	∆PCE (%)	Stability time/condition	Proposed strategy	References
Mesoporous/Spiro-OMeTAD	13.3	47	550 h (RH = 40%)	Durability and scalability	^24^
Inverted planar/PEDOT: PSS	14.35	22	56 days (in the glove box)	Self-restraint	^20^
Planar/Spiro-OMeTAD + MoO_3_	20.18	30	300 h (ambient)	Flower-like MoS_2_	^26^
Mesoporous/Spiro-OMeTAD	14.34	2	1 h (ambient)	MoS_2_ annealing method variation	^28^
Mesoporous/Spiro-OMeTAD	9.5	45	120 h (RH = 40–50%)	MoS_2_ concentration variation	This work

## Conclusion

This work established the incorporation of a few-layer MoS_2_ as hybrid HTL in the PSC structure. Different concentrations of MoS_2_ dispersion from 0 to 4.0 mg mL^−1^ were successfully deposited via the facile spin coating method. The incorporation of MoS_2_ could enhance the charge transfer and hole mobility of spiro-OMeTAD at 2.0 mg mL^−1^ optimum concentration resulting in PCE of 9.5%. The layer could stifle the lithium-ion migration from the spiro-OMeTAD to the perovskite layer. Remarkably, the incorporation of MoS_2_ mainly prolonged the film stability by maintaining the degradation percentage of 45% of initial PCE after 120 h aging with the device remained unencapsulated. However, further studies on the precise number of layers for each concentration are obligatory for the realization of excellent PCE and stability of PSC in the future.

## Supplementary Information

Supplementary Information.

## Data Availability

All data that support these findings are included within this article and Supplementary Materials.
