# Supplementary material for: Stabilizing high-humidity perovskite solar cells with MoS2 hybrid HTL
Source: Sci Rep. 2023 Jul 25;13:11996. doi: 10.1038/s41598-023-39189-0 (PMC10368666; doi:10.1038/s41598-023-39189-0)
Supplement: Supplementary file 1 — Supplementary Information. [file 41598_2023_39189_MOESM1_ESM.docx]

**Stabilizing High-Humidity Perovskite Solar Cells with MoS_2_ Hybrid HTL**

Puteri Nor Aznie Fahsyar ^1,2*^, Norasikin Ahmad Ludin^2*^, Noor Fadhilah Ramli^2^, Puteri Intan Zulaikha^2^, Suhaila Sepeai^2^, Ahmad Shah Hizam Md Yasir^3^

^1^Clean Technology Impact Laboratory, Taylor’s University, Selangor, Malaysia

^2^Solar Energy Research Institute, University Kebangsaan Malaysia, Selangor, Malaysia

^3^Faculty of Resilience, Rabdan Academy, Abu Dhabi, United Arab Emirates

*Corresponding authors: puteri.syedmahadzir@taylors.edu.my; [sheekeen@ukm.edu.my](mailto:%20sheekeen@ukm.edu.my)

**Supplementary documents**


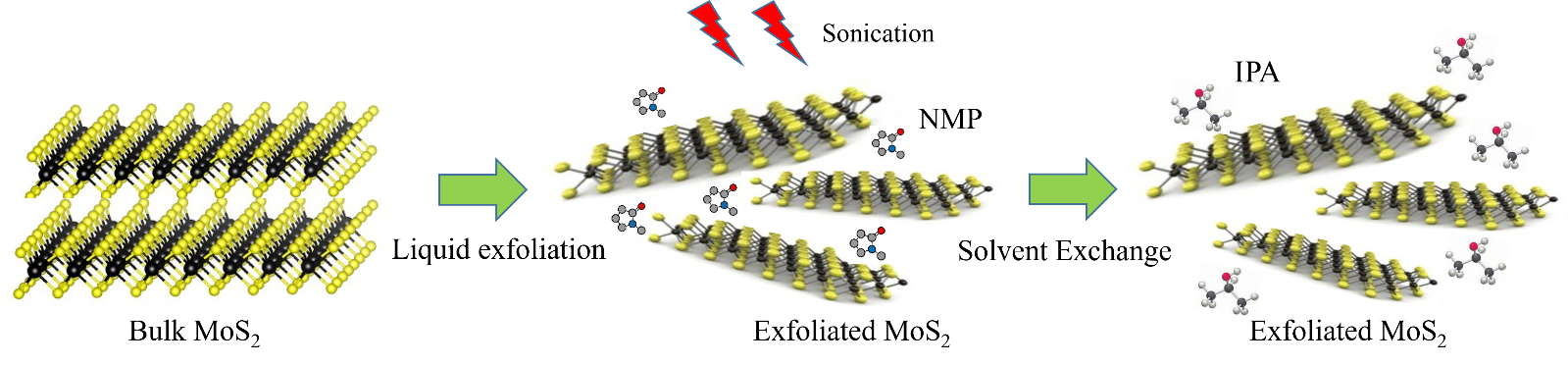


**Fig. S1**. MoS_2_ preparation using liquid exfoliation and solvent exchange process.


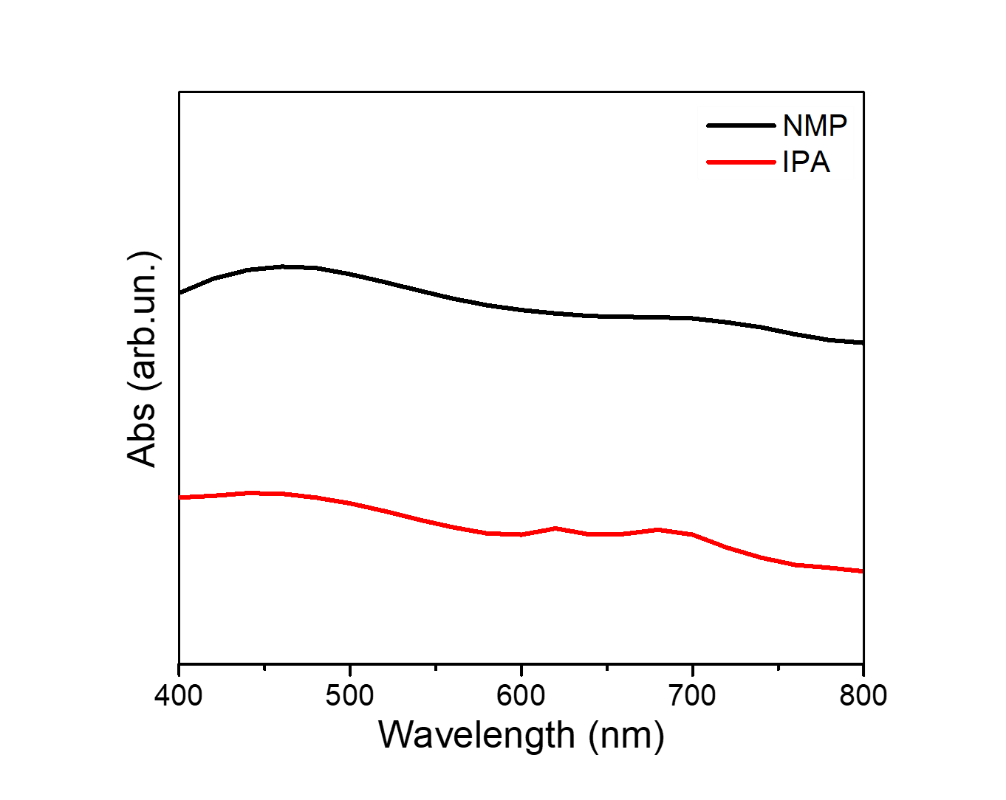


**Fig. S2.** Absorbance spectra of exfoliated MoS_2_ in NMP and IPA

**Table S1.** The values of all parameters of Raman peak shift.

| Sample | E12g (cm-1) | A1g (cm-1) | A1g -E12g (cm-1) | Thickness(nm) |
| --- | --- | --- | --- | --- |
| M1 | 381.6 | 404.6 | 23.0 | 9 |
| M2 | 381.1 | 405.1 | 24.0 | 29 |
| M3 | 380.6 | 405.6 | 25.0 | 45 |
| M4 | 380.1 | 406.5 | 26.4 | 65 |

**Table S2.** Summary of the bandgap for different concentrations of MoS_2._

| Sample | Eg, eV |
| --- | --- |
| M0 | 1.59 |
| M1 | 1.59 |
| M2 | 1.58 |
| M3 | 1.60 |
| M4 | 1.61 |

**Table S3.** The values of all parameters of the fitted curves in the Nyquist plot.

| Sample | R_S_ (Ω) | CPE_1_ (F) | R_CT_ (Ω) | CPE_2_ (F) | R_Rec_ (Ω) |
| --- | --- | --- | --- | --- | --- |
| M0 | 72.67 | 7.948x10^-7^ | 223.27 | 5.440x10^-5^ | 368.7 |
| M1 | 62.46 | 9.159x10^-7^ | 280.7 | 9.101x10^-4^ | 1026 |
| M2 | 45.81 | 2.310x10^-7^ | 183.4 | 1.720x10^-4^ | 3983.5 |
| M3 | 111.22 | 6.847x10^-8^ | 390.4 | 5.430x10^-5^ | 874.6 |
| M4 | 145.58 | 1.184x10^-6^ | 588.56 | 1.523x10^-4^ | 5944 |

**Table S4.** Photovoltaic-performance parameters for M0 samples under 1 sun illumination (AM 1.5G, 100 mW cm^−2^) for PSC device at reverse scan direction.

| Time (hours) | *J_sc_* (mA/cm^2^) | *V_oc_* (V) | *FF* | PCE (%) |
| --- | --- | --- | --- | --- |
| 0 | 16.6 | 0.92 | 0.54 | 8.3 |
| 24 | 15.8 | 0.83 | 0.54 | 7.1 |
| 48 | 15.0 | 0.80 | 0.52 | 6.3 |
| 72 | 14.3 | 0.77 | 0.38 | 4.2 |
| 96 | 10.6 | 0.70 | 0.27 | 2.0 |
| 120 | 8.1 | 0.61 | 0.24 | 1.2 |

**Table S5.** Photovoltaic-performance parameters for M1 samples under 1 sun illumination (AM 1.5G, 100 mW cm^−2^) for PSC device at reverse scan direction.

| Time (hours) | *J_sc_* (mA/cm^2^) | *V_oc_* (V) | *FF* | PCE (%) |
| --- | --- | --- | --- | --- |
| 0 | 16.8 | 0.94 | 0.54 | 8.7 |
| 24 | 16.0 | 0.89 | 0.53 | 7.5 |
| 48 | 15.6 | 0.82 | 0.53 | 6.8 |
| 72 | 13.8 | 0.76 | 0.50 | 5.1 |
| 96 | 13.1 | 0.72 | 0.49 | 4.7 |
| 120 | 10.6 | 0.66 | 0.46 | 3.2 |

**Table S6.** Photovoltaic-performance parameters for M2 samples under 1 sun illumination (AM 1.5G, 100 mW cm^−2^) for PSC device at reverse scan direction.

| Time (hours) | *J_sc_* (mA/cm^2^) | *V_oc_* (V) | *FF* | PCE (%) |
| --- | --- | --- | --- | --- |
| 0 | 17.2 | 0.97 | 0.57 | 9.5 |
| 24 | 16.8 | 0.91 | 0.57 | 8.7 |
| 48 | 15.7 | 0.87 | 0.57 | 7.9 |
| 72 | 15.2 | 0.82 | 0.57 | 7.1 |
| 96 | 14.8 | 0.78 | 0.56 | 6.5 |
| 120 | 14.0 | 0.68 | 0.55 | 5.2 |

**Table S7.** Photovoltaic-performance parameters for M3 samples under 1 sun illumination (AM 1.5G, 100 mW cm^−2^) for PSC device at reverse scan direction.

| Time (hours) | *J_sc_* (mA/cm^2^) | *V_oc_* (V) | *FF* | PCE (%) |
| --- | --- | --- | --- | --- |
| 0 | 14.5 | 0.97 | 0.53 | 7.6 |
| 24 | 13.7 | 0.95 | 0.53 | 7.0 |
| 48 | 12.6 | 0.92 | 0.51 | 5.9 |
| 72 | 11.5 | 0.88 | 0.49 | 5.0 |
| 96 | 11.2 | 0.86 | 0.49 | 4.9 |
| 120 | 10.8 | 0.81 | 0.49 | 4.6 |

**Table S8.** Photovoltaic-performance parameters for M4 samples under 1 sun illumination (AM 1.5G, 100 mW cm^−2^) for PSC device at reverse scan direction.

| Time (hours) | *J_sc_* (mA/cm^2^) | *V_oc_* (V) | *FF* | PCE (%) |
| --- | --- | --- | --- | --- |
| 0 | 14.2 | 0.97 | 0.50 | 6.9 |
| 24 | 13.1 | 0.94 | 0.49 | 6.0 |
| 48 | 12.4 | 0.90 | 0.47 | 5.1 |
| 72 | 11.9 | 0.87 | 0.47 | 4.9 |
| 96 | 10.9 | 0.85 | 0.47 | 4.5 |
| 120 | 10.5 | 0.84 | 0.47 | 4.3 |
